# Monkeypox self-diagnosis abilities, determinants of vaccination and self-isolation intention after diagnosis among MSM, the Netherlands, July 2022

**DOI:** 10.2807/1560-7917.ES.2022.27.33.2200603

**Published:** 2022-08-18

**Authors:** Haoyi Wang, Kennedy J.I. d'Abreu de Paulo, Thomas Gültzow, Hanne M.L. Zimmermann, Kai. J. Jonas

**Affiliations:** 1 Department of Work and Social Psychology, Maastricht University, Maastricht, The Netherlands

**Keywords:** Monkeypox, Vaccination, MSM, Prevention, Self-diagnosis, Isolation

## Abstract

Monkeypox is a zoonotic disease and leads to a smallpox-like disease in humans. The current epidemic in European countries requires informed responses. We investigated the ability to self-diagnose a potential infection, and determinants of vaccination and self-isolation intention after diagnosis among 394 MSM in the Netherlands. We found that about half were able to self-diagnose monkeypox, that 70% had a high intention to get vaccinated and 44% to self-isolate after monkeypox diagnosis. Determinants went beyond mere risk behaviour criteria.

The global epidemic of monkeypox, a zoonotic disease, has recently changed from infections predominantly following an interaction with animals, to human-to-human transmission [1,2]. The monkeypox epidemic in European countries is currently predominantly affecting men-who-have-sex-with-men (MSM) and the number of infections is still increasing [3,4]. In the Netherlands 1,025 cases have been reported as at 11 August 2022, the majority in Amsterdam. Given the spread of the disease, investigating self-diagnostic abilities, and vaccination and self-isolation intentions is relevant to tailor further public health responses.

## Survey and participants

We conducted an online survey among MSM using a cohort established in 2017 [5] (n = 257), along with recruitment of MSM on a gay online dating app (n = 137) in the first half of July 2022, before the start of targeted monkeypox vaccination in the Netherlands [6]. Of the included 394 MSM, 43% (n=171) were below the age of 45-years, 6% (n=22) were living with HIV and 66% (n=241) were currently using HIV pre-exposure prophylaxis (PrEP) (see supplementary materials S1 for the population characteristics by PrEP use status, and S2 for participants’ age distribution).

## Monkeypox self-diagnosis

We provided participants with four images and asked them to indicate what condition this could be. All of the images were showing lesions in parts of the face, one depicted a monkeypox lesion, the other three a vesicle due to a staphylococcal skin infection, a syphilis stage-2, and eczema. Only the image of eczema was diagnosed predominantly correct (67%), monkeypox lesion and staphylococcal infection images triggered some correct hits, but also considerable amounts of false self-diagnoses (up to 48% and 58%, respectively, for all alternative diagnoses combined), syphilis stage-2 was most frequently misdiagnosed as monkeypox (52%) (Table 1).

**Table 1:** Ability to self-diagnose a monkeypox lesion among MSM, the Netherlands July 2022 (n=394)

Displayed on image	Self-diagnosis							
	Monkeypox		Staphylococcus		Syphilis		Eczema	
	n	%	n	%	n	%	n	%
**Monkeypox**	206	52.3	150	38.1	30	7.6	8	2.0
**Staphylococcal skin infection**	94	23.9	167	42.4	105	26.6	28	7.1
**Syphilis stage-2**	204	51.8	91	23.1	91	23.1	8	2.0
**Eczema**	14	3.6	98	24.9	20	5.1	262	66.5

## Vaccination and self-isolation intentions

Vaccination intention and self-isolation intention were measured on a 1─5 Likert scale (with 1=“Very low” and 5=“Very high”). In this study, we categorised these two endpoints as “High/very high (scale 4 and 5)” and “The rest of the scale” for the modelling analysis. Overall, 70% of participants showed high vaccination intention and 44% showed high intention for self-isolation after diagnosis i.e. until all lesions are gone, usually for up to 21 days [7]. Given that currently monkeypox vaccinations are administered to PrEP-using MSM in the Netherlands [8], we adjusted for PrEP use status (current users (n = 241) vs PrEP-naïve MSM or PrEP-discontinued MSM (n = 122)) to compare the standardised prevalence ratio (SPR). We found that despite of the higher prevalence among PrEP users in both vaccination intention and self-isolation intention, the adjusted SPRs showed no significant differences, indicating similar vaccination and self-isolation intentions among PrEP users and non-PrEP-users (Table 2).

**Table 2:** Prevalence and standardised prevalence ratio of monkeypox vaccination and self-isolation intention, the Netherlands, July 2022 (n=394)

Sub-population	Vaccination intention (Extremely likely vs rest of scale)^a^					Monkeypox self-isolation intention (Extremely likely vs rest of scale)^a^				
	Prevalence		95%CI	SPR^b^	95%CI	Prevalence		95%CI	SPR^b^	95%CI
	n	%				n	%			
**PrEP users**	178	73.86	67.97–79.00	1.01	0.87–1.17	105	43.57	37.45–49.81	1.06	0.87–1.28
**Non-PrEP users**	87	71.31	62.73–78.59	0.97	0.78–1.19	43	35.25	27.34–33.06	0.86	0.63–1.14
**Total sample**	276	70.01	65.35–74.36	NA	NA	173	43.91	39.09–48.84	NA	NA

CI: confidence interval; NA: not applicable; PrEP: pre-exposure prophylaxis; SPR: standardised prevalence ratio.

^a^ 1−5 Likert scale, with 1 = extremely unlikely and 5 = extremely likely)

^b^ Adjusted for PrEP use status.

## Determinants predicting vaccination and self-isolation intention

To identify vaccination intention differences and among MSM sub-populations and which sub-population may follow the current self-isolation policy best, we conducted two multivariable logistic regression analyses with socio-demographic, behavioural, and psychosocial determinants. First, we conducted univariable logistic modelling with each of the selected determinants to investigate potential correlations with being extremely likely to get vaccinated. We retained all determinants with p < 0.10 in the univariable analysis with the full variance in the multivariable model, given the relatively small sample size (Table 3) (supplementary materials S3 shows the analysis for collinearity). In a sensitivity analysis, we combined somewhat- and extremely-likely intentions to define high intention to explore the implications on the endpoint selection (sensitivity analysis results are shown in the supplementary materials S4).

**Table 3:** Determinants of monkeypox vaccination intention and self-isolation intention, the Netherlands, July 2022 (n=394)

Variables	Vaccination intention (extremely likely vs rest of scale)						Self-isolation intention (extremely likely vs rest of scale)					
	Univariable model			Multivariable model			Univariable model			Multivariable model		
	OR	95%CI	p value	aOR	95%CI	p value	OR	95%CI	p value	aOR	95%CI	p value
**Socio-demographic determinants**												
**Age**												
< 45 years	Ref.			NA			Ref.			Ref.		
> 45 years	1.04	0.67─1.61	0.861				1.41	0.94─2.11	0.098	1.11	0.70─1.77	0.663
**Relationship**												
Single	Ref.			Ref.			Ref.			Ref.		
Single but dating	1.98	1.06─3.73	0.033	2.42	1.13─5.20	0.024	1.84	0.99─3.41	0.052	1.57	0.82─3.03	0.177
Monogamous relationship	0.79	0.36─1.75	0.564	1.25	0.45─3.51	0.671	0.79	0.34─1.85	0.586	0.70	0.28─1.72	0.434
Open/polyamorous relationship	3.44	1.94─6.08	< 0.001	3.96	1.97─7.99	0.001	1.44	0.84─2.47	0.186	1.20	0.67─2.17	0.536
**Education**												
Lower than bachelor	Ref.			NA			Ref.			Ref.		
Bachelor	0.94	0.53─1.67	0.832				0.51	0.30─0.89	0.017	0.54	0.30─0.95	0.034
Master	1.17	0.66─2.08	0.593				0.49	0.29─0.84	0.009	0.52	0.29─0.93	0.029
PhD or higher	1.91	0.71─5.18	0.202				0.73	0.32─1.66	0.454	0.83	0.34─2.56	0.676
**Employment**												
Employed	Ref.			Ref.			Ref.			Ref.		
Unemployed or receiving social welfare	0.84	0.53─1.67	0.733	0.99	0.29─3.48	0.991	2.01	0.75─5.42	0.166	1.77	0.63─4.93	0.274
Retired	9.58	0.66─2.08	0.028	11.04	1.35─90.36	0.025	4.79	1.73─13.30	0.003	5.35	1.84─15.57	0.002
Student	1.06	0.71─5.18	0.899	1.32	0.44─3.99	0.613	0.76	0.30─1.95	0.566	0.94	0.34─2.56	0.901
**Migration status**												
Not applicable	Ref.			NA			Ref.			Ref.		
First-generation migrant	0.83	0.44─1.55	0.552				1.74	0.96─3.14	0.069	1.82	0.97─3.45	0.064
Second-generation migrant	0.90	0.31─2.69	0.863				2.38	0.84─6.69	0.101	2.59	0.84─8.00	0.098
**Residence**												
The rest of the country	Ref.			NA			Ref.			NA		
Randstad (main urban area)	1.34	0.87─2.08	0.186				0.79	0.53─1.19	0.263			
**Behavioural determinants**												
**Number of sex partners in the previous 6 months**												
None	Ref.			Ref.			Ref.			NA		
1	2.36	0.50─11.12	0.275	1.70	0.23─12.63	0.603	0.71	0.15─3.35	0.669			
2─6	3.33	0.76─14.48	0.108	1.62	0.24─1099	0.617	0.45	0.10─1.94	0.283			
7─15	5.21	1.16─23.42	0.031	2.26	0.32─16.25	0.415	0.35	0.08─1.59	0.176			
More than 15	6.37	1.38─29.36	0.018	2.02	0.26─15.39	0.499	0.52	0.12─2.31	0.389			
**HIV status**												
HIV-negative	Ref.			NA			Ref.			NA		
HIV-positive	1.11	0.42─2.93	0.826				1.31	0.55─3.10	0.536			
HIV status unknown or not disclosed	0.33	0.08─1.27	0.107				1.64	0.43─6.21	0.466			
**PrEP use status**												
Current PrEP user	Ref.			Ref.			Ref.			NA		
PrEP naïve or PrEP discontinued	0.66	0.41─1.06	0.0872	0.78	0.41─1.49	0.447	0.79	0.50─1.22	0.286			
**Any type of substance use in the previous 6 months**												
Never	Ref.			NA			Ref.			NA		
Ever	0.60	0.32─1.14	0.121				1.39	0.74─2.58	0.302			
**Recreational drugs use in the previous 6 months ^a^**												
Never	Ref.			NA			Ref.			NA		
Ever	*1.39*	0.88─2.19	0.163				*0.95*	0.63─1.43	0.7957			
**Chemsex in the previous 6 months ^b^**												
Never	Ref.			NA			Ref.			NA		
Ever	0.90	0.55─1.46	0.659				0.88	0.56─1.39	0.585			
**Poppers use in the previous 6 months**												
Never	Ref.			NA			Ref.			NA		
Ever	1.35	0.88─2.08	0.173				1.10	0.73─1.64	0.632			
**Erectile dysfunction medication use in the previous 6 months ^c^**												
Never	Ref.			Ref.			Ref.			NA		
Ever	1.48	0.95─2.30	0.087	0.77	0.43─1.39	0.380	1.35	0.90─2.02	0.144			
**Alcohol use in the previous 6 months**												
Never	Ref.			NA			Ref.			NA		
Ever	1.08	0.65─1.77	0.766				0.79	0.49─1.26	0.320			
**Visited a gay sauna in the previous 6 months**												
Never	Ref.			NA			Ref.			NA		
Ever	1.44	0.91─2.28	0.119				1,01	0.66─152	0.965			
**Visited a darkroom in the previous 6 months**												
Never	Ref.			NA			Ref.			NA		
Ever	1.34	0.86─2.10	0.199				1.32	0.88─1.98	0.178			
**Visited a circuit party in the previous 6 months**												
Never	Ref.			NA			Ref.			NA		
Ever	1.01	0.63─1.64	0.952				1.24	0.80─1.93	0.337			
**Visited a pride event in the previous 6 months**												
Never	Ref.			NA			Ref.			NA		
Ever	1.17	0.76─1.80	0.481				1.01	0.68─1.50	0.978			
**Visited a gay dance club in the previous 6 months**												
Never	Ref.			NA			Ref.			NA		
Ever	1.05	0.67─1.65	0.823				1.05	0.69─1.60	0.806			
**Attended private sex parties in the previous 6 months**												
Never	Ref.			NA			Ref.			NA		
Ever	1.20	0.74─1.93	0.464				1.20	0.77─1.85	0.421			
**Visited fetish events/fairs in the previous 6 months**												
Never	Ref.			NA			Ref.			NA		
Ever	1.06	0.62─1.78	0.840				1.21	0.75─1.96	0.427			
**Psychosocial determinants**												
**Knowing anybody who has/had monkeypox**												
No	Ref.			Ref.			Ref.			NA		
Yes	2.87	1.41─5.84	0.003	2.33	0.98─5.52	0.055	1.17	0.69─1.97	0.565			
**Concern about being infected by monkeypox^d^**	1.74	1.40─2.16	< 0.001	1.74	1.35─2.26	< 0.001	1.10	0.94─1.30	0.236			
**Perceived risk of being infected by monkeypox^d^**	1.73	1.40─2.13	< 0.001	1.21	0.88─1.64	0.237	1.13	0.94─1.36	0.190			
**Perceived problematic consequences of monkeypox^d^**	1.21	0.98─1.50	0.075	1.18	0.90─1.53	0.224	1.35	1.09─1.66	0.005	1.39	1.11─1.74	0.005

aOR: adjusted odds ratio; CI: confidence interval; GHB: gamma-hydroxybutyric acid; MDMA: 3,4-methylenedioxymethamphetamine; NA: not applicable; OR: odds ratio; PrEP: pre-exposure prophylaxis; THC: tetrahydrocannabinol.

^a^ I use substances recreationally (for example THC, MDMA, ecstasy, etc).

^b^ I use substances in the context of sex (for example crystal methamphetamine/tina, GHB, ketamine etc.).

^c^ I use erectile dysfunction medication (for example Viagra, Kamagra).

^d^ indicates variable with a 1−5 Likert scale, with 1 = extremely unlikely and 5 = extremely likely)

For vaccination intention as endpoint, among socio-demographic determinants, MSM who were single but dating (adjusted OR (aOR) = 2.42), who had an open/polyamorous relationship (aOR = 3.96) and who were retired (aOR = 11.04) were more likely to have high vaccination intentions. No behavioural determinants were found to be statistically associated. Among psycho-social determinants, we found that knowing someone who has/had monkeypox (aOR = 2.33) and being worried about a monkeypox infection (aOR = 1.74) was associated with high vaccination intention.

For self-isolation intention as endpoint, almost all included socio-demographic determinants showed relevant associations. MSM with bachelor (aOR = 0.54) and master (aOR = 0.52) degrees were less likely to self-isolate after diagnosis for 21 days. Those who were retired (aOR = 5.35) showed higher intentions on the other hand. Similar to the vaccination intention, no behavioural determinant was found to be associated with high self-isolation intentions. Among psychosocial determinants, MSM who perceived more problematic consequences because of a monkeypox infection (aOR = 1.39) were more likely to self-isolate.

## Discussion

Given the incubation period of 9 days in the case of exposure via mucous membranes (e.g., anus, mouth, urethra) [9], it is important to detect monkeypox infections quickly to limit onward transmission, which highlights the importance of affected individuals’ ability to detect the typical lesions early on. The findings from a survey including 394 MSM in the Netherlands showed that only about half of them were able to self-diagnose monkeypox from other skin lesions which shows that self-diagnosis in particular of novel diseases, can be challenging. Furthermore, infected individuals should isolate for up to 21 days until all symptoms are gone [4]. Especially after all coronavirus disease (COVID-19)-related quarantine measures, there are limited data available on the acceptance of such measures for other diseases, such as monkeypox. Our results showed that only 44% intended to fully self-isolate after receiving a monkeypox diagnosis.

At the same time, vaccination programs have commenced in many countries that focus on high-risk populations, for example MSM who are using PrEP [8]. Our results revealed that 70% belonging to this group had a high intention to get vaccinated. Given vaccine scarcity [10], it is paramount to identify the populations with the highest risk, but also to gauge general vaccination intention in moderate at-risk populations.

Our main findings hold relevant implications to guide national response to the monkeypox outbreak. Firstly, both the ability to self-diagnose and intention to self-isolate were relatively low among our study participants. Public health and social measures are considered essential [11] to reduce transmission of pathogens with epidemic potential. Thus, health education campaigns/interventions should not only aim to increase vaccination intentions, but also to support concerned individuals in following other mitigation measures such as isolation. Secondly, although close contact during sexual activities is the most frequently suspected route of infection [12] and the main reason to prioritise MSM using PrEP for vaccination in the Netherlands since 25 July 2022 [6], high intention to get vaccinated and self-isolate in this study were not predicted by any of the sexual risk behaviours. This is of note, as the current public communication and vaccination strategy in the Netherlands focuses on motivating those showing higher sexual risk behaviours, while in our study socio-demographic (e.g., education level and migration status) and psycho-social variables (e.g., “knowing someone that has/had monkeypox” and “higher concern about getting infected by monkeypox”) were found predictive for a higher vaccination and self-isolation intention. Our results could inform campaign content and dissemination planning of respective monkeypox prevention campaigns. They suggest it is crucial to increase our efforts to also reach highly educated with these messages, as well as to present realistic stories of monkeypox infections using peer-communication without inducing fear. Even though our findings and other research imply that under certain conditions having fear could increase intention to vaccinate and self-isolate [13].

Also, since vaccination intentions did not differ between PrEP users and non-PrEP-users and non-PrEP-users may also have risk behaviours, prioritising PrEP users may not be sufficient to reach the full potential of the vaccination. Finally, albeit most Dutch monkeypox cases have been reported from Amsterdam, our analysis of geographic differences did not yield significant results.

However, our study is not devoid of limitations. As our analyses were conducted based on MSM from an existing cohort which aimed to investigate PrEP use [9] and MSM who were on an online gay dating app, both groups of participants may be more familiar with new media and rapid access to information sources, and thus may be more likely to have more knowledge about the monkeypox epidemic compared with the general MSM population living in the Netherlands. This may result in overestimated parameters. Additionally, PrEP use may be overrepresented among our participants compared with the general MSM population given the sampling strategy and hence our results may be limited to this population. Yet at the same time, PrEP users are the target group for monkeypox vaccinations in the Netherlands at the moment. Thus, we believe that our sample is capturing relevant parts of the at-risk population. Furthermore, we assessed the concern of getting a monkeypox infection, but did not investigate its determinants further. We believe that future research should investigate preventions intentions, for example, those that reflect changes in vaccine availability, and complement the emerging picture on attitudes related to monkeypox, such as determinants of infection concerns, and also triangulate results obtained among MSM with healthcare provider perspectives. Data from other contexts, for example Italy, show that healthcare providers currently lack relevant knowledge, underestimate the epidemic and the need for tailored responses [14].

## Conclusions

To conclude, based on our findings public health policy makers and services should in addition to focussing on vaccinations in their communication, aim to increase MSM’s intentions to self-isolate and their ability to accurately self-diagnose. Efforts should also be stepped up to target MSM at the highest risk, especially those with little concern about monkeypox and those with a high level of education. Even though obtained in a Dutch context, we believe our findings may also be relevant for other countries with similar conditions.

## Supplementary Data

496000 Supplementary Materials
