# Supplementary material for: Monkeypox self-diagnosis abilities, determinants of vaccination and self-isolation intention after diagnosis among MSM, the Netherlands, July 2022
Source: Euro Surveill. 2022 Aug 18;27(33):2200603. doi: 10.2807/1560-7917.ES.2022.27.33.2200603 (PMC9389856; doi:10.2807/1560-7917.ES.2022.27.33.2200603)
Supplement: Supplementary Materials [file 22-00603_JONAS_SupplementaryMaterials.pdf]

Supplementary materials

"This supplementary material is hosted by *Eurosurveillance* as supporting information alongside the article [Monkeypox self-diagnosis abilities, determinants of vaccination intention and self-isolation intention after diagnosis among MSM in the Netherlands], on behalf of the authors, who remain responsible for the accuracy and appropriateness of the content. The same standards for ethics, copyright, attributions and permissions as for the article apply. Supplements are not edited by *Eurosurveillance* and the journal is not responsible for the maintenance of any links or email addresses provided therein."

Table S1. Study characteristics by total sample and by PrEP use status.

Variables			Total sample (n=394)		PrEP user (n=241)		Non-PrEP user (n=122)		Chi-square test (p-value)
			N	%	N	%	N	%	
Socio-demographic determinants	Age								
		<45 years	171	43.4	90	37.34	69	56.56	<.001
		>45 years	223	56.6	151	62.66	53	43.44	
	Relationship								
		Single	79	20.05	47	19.5	27	22.13	<.001
		Single but dating	91	23.1	58	24.07	26	21.31	
		Monogamous relationship	35	8.88	6	2.49	27	22.13	
		Open/Polyamorous relationship	189	47.97	130	53.94	42	34.43	
	Education								
		Lower than Bachelor	89	22.65	62	25.73	19	15.57	.125
		Bachelor	131	33.33	80	22.1	41	33.61	
		Master	142	36.13	81	22.38	50	40.98	
		PhD or higher	31	7.89	17	4.7	12	9.84	
	Employment								
		Employed	335	85.03	208	86.31	102	83.61	.602
		Unemployed or receiving social welfare	22	5.58	10	4.15	4	3.28	
		Retired	20	5.08	13	5.39	7	5.74	
		Student	17	4.31	10	4.15	9	7.38	
	Migration status								
		Not applicable	325	82.91	200	82.99	101	82.79	.426
		First-generation migrant	51	13.01	29	12.03	18	14.75	
		Second-generation migrant	16	4.08	12	4.98	3	2.46	
	Residence								
		The rest of the country	154	39.10	95	39.42	49	40.16	.891

		Randstad (main urban area)	240	60.90	146	60.58	73	59.84	
Behavioural determinants	Number of sex partners in the previous 6 months								
		None	8	2.03	1	0.41	6	4.92	<.001
		1	46	11.68	13	5.39	28	22.95	
		2 to 6	82	20.81	66	27.39	10	8.2	
		7 to 15	159	40.36	91	37.76	59	48.36	
		More than 15	99	25.13	70	29.05	19	15.57	
	HIV status								
		HIV-negative	363	92.13	241	100,00	122	100,00	NA
		HIV-positive	22	5.58	0	0,00	0	0,00	
		HIV status unknown or not disclosed	9	2.28	0	0,00	0	0,00	
	PrEP use status								
		Current PrEP users	241	66.39	NA	NA	NA	NA	NA
		PrEP naïve or PrEP discontinued	122	30.96	NA	NA	NA	NA	
	Any type of substance use in the previous 6 months								
		Never	349	11.42	221	91.7	102	83.61	.020
		Ever	45	11.42	20	8.3	20	16.39	
	Recreational drugs use in the previous 6 months					0,00		0,00	
		Never	250	63.45	142	58.92	88	72.13	.014
		Ever	144	36.55	99	41.08	34	27.87	
	Chemsex in the previous 6 months								
		Never	293	74.37	152	63.07	115	94.26	<.001
		Ever	101	25.63	89	36.93	7	5.74	
	Poppers use in the previous 6 months								
		Never	183	46.45	88	36.51	82	67.21	<.001
		Ever	211	53.55	153	63.49	40	32.79	
	Erectile dysfunction medication use in the previous 6 months								

	Never	228	57.87	110	45.64	99	81.15	<.001
	Ever	166	42.13	131	54.36	23	18.85	
Alcohol use in the previous 6 months								
	Never	93	23.6	57	23.65	29	23.77	.980
	Ever	301	76.4	184	76.35	93	76.23	
Visited a gay sauna in the previous 6 months								
	Never	251	63.71	142	58.92	89	72.95	.009
	Ever	143	36.29	99	41.08	33	27.05	
Visited a darkroom in the previous 6 months								
	Never	238	60.41	127	52.7	90	73.77	<.001
	Ever	156	39.59	114	47.3	32	26.23	
Visited a circuit party in the previous 6 months								
	Never	283	71.83	162	67.22	96	78.69	.023
	Ever	111	28.17	79	32.78	26	21.31	
Visited a pride event in the previous 6 months								
	Never	203	51.52	119	49.38	63	51.64	.684
	Ever	191	48.48	122	50.62	59	48.36	
Visited a gay dance club in the previous 6 months								
	Never	137	34.77	74	30.71	48	39.34	.100
	Ever	257	65.23	167	69.29	74	60.66	
Attended private sex parties in the previous 6 months								
	Never	277	70.3	145	60.17	110	90.16	<.001
	Ever	117	29.7	96	39.83	12	9.84	
Visited fetish events/fairs in the previous 6 months								
	Never	308	78.17	174	72.2	106	86.89	.002
	Ever	86	21.83	67	27.8	16	13.11	
Knowing anybody who has/had monkeypox								

	No	326	82.74	193	80.08	107	87.7	.070
	Yes	68	17.26	48	19.92	122	100,00	
Psycho-social determinants	Concern about being infected by monkeypox*	4	[2-4]	4	[2-4]	4	[2-4]	0.043
	Perceived risk of being infected by monkeypox*	3	[2-4]	3	[2-4]	3	[2-4]	0.026
	Perceived problematic consequences of monkeypox*	4	[3-4]	4	[3-4]	4	[3-5]	0.206

Notes: [1] I use substances recreationally (for example THC, MDMA, ecstasy, etc). [2] I use substances in the context of sex (for example crystal meth/tina, GHB, ketamine etc.). [3] I use erectile dysfunction medication (for example Viagra, Kamagra). * indicates variable with a 1−5 Likert scale, with 1 = extremely unlikely and 5 = extremely likely), results were reported in median [interquartile range]. NA = not applicable.

Table S2. Participant age distribution

Age group	Total sample (n=394)		PrEP user (n=241)		Non-PrEP user (n=122)	
	N	%	N	%	N	%
16-18	2	0.5	1	0.4	0	0.0
18-24	11	2.8	2	0.8	8	3.3
25-34	91	23.1	49	20.3	39	16.2
35-44	67	17.0	38	15.8	22	9.1
45-54	143	36.3	98	40.7	30	12.4
55-64	58	14.7	40	16.6	16	6.6
65-74	21	5.3	12	5.0	7	2.9
75 and older	1	0.2	1	0.4	0	0.0

Figure S3 Collinearity analysis for included variables

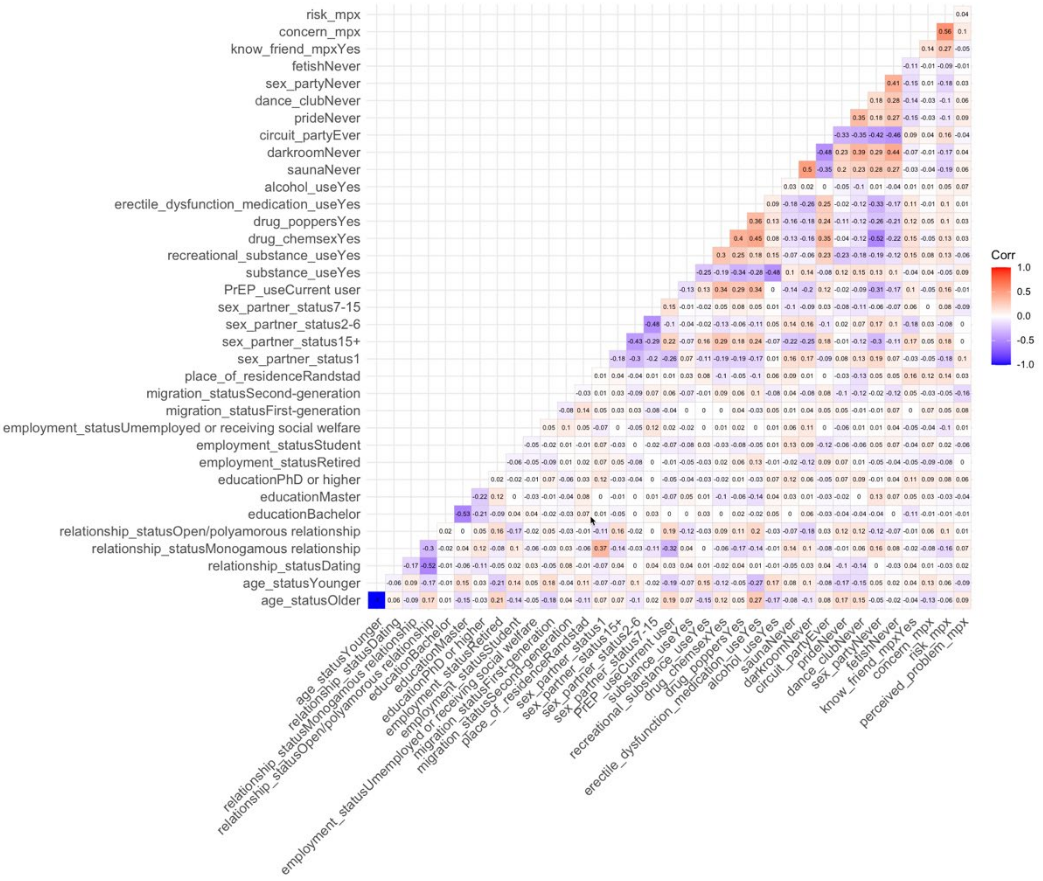

Table S4. Sensitivity analysis - determinants of Monkeypox vaccination intention and self-isolation intention

Variables		Vaccination intention (Somehow and extremely likely vs. rest of scale)						Self-isolation intention (Somehow and extremely likely vs. rest of scale)					
		Univariable model			Multivariable model			Univariable model			Multivariable model		
		OR	95%CI	p-value	aOR	95%CI	p-value	OR	95%CI	p-value	aOR	95%CI	p-value
**Socio-demographic determinants**	Age												
	<45 years	ref.	-	-				ref.	-	-			
	>45 years	1.12	0.57;2.21	0.743				0.84	0.52;1.34	0.461			
	Relationship												
	Single	ref.	-	-	ref.	-	-	ref.	-	-			
	Single but dating	2.54	0.90;7.11	0.077	2.66	0.87;8.11	0.078	0.83	0.41;1.69	0.609			
	Monogamous relationship	0.72	0.26;2.01	0.526	0.98	0.30;3.13	0.998	0.87	0.34;2.23	0.779			
	Open/Polyamorous relationship	2.64	1.13;6.17	0.025	2.42	0.96;6.06	0.047	0.90	0.49;1.65	0.722			
	Education												
	Lower than Bachelor	ref.	-	-				ref.	-	-			
	Bachelor	1.40	0.59;3.32	0.448				0.79	0.42;1.50	0.473			
	Master	1.40	0.60;3.28	0.439				1.03	0.56;1.91	0.914			
	PhD or higher	4.23	0.52;34.20	0.176				0.89	0.34;2.43	0.811			
	Employment												
	Employed	ref.	-	-				ref.	-	-	ref.	-	-
	Unemployed or receiving social welfare	0.82	0.18;3.74	0.797				1.31	0.45;3.82	0.625	1.32	0.45;3.88	0.613
	Retired	464836.00	0.00;inf	0.985				0.15	0.02;1.13	0.065	0.15	0.02;1.13	0.066
	Student	0.98	0.22;4.43	0.987				1.04	0.37;2.96	0.933	1.01	0.25;2.88	0.986
	Migration status												
	Not applicable	ref.	-	-				ref.	-	-			
	First-generation migrant	0.53	0.23;1.23	0.137				0.56	0.25;1.24	0.154			

	Second-generation migrant	416842.00	0.00;inf	0.988	0.43	0.10;1.93	0.271
	Residence						
	The rest of the country	ref.	-	-	ref.	-	-
	Randstad (main urban area)	1.54	0.78;3.03	0.213	0.94	0.58;1.51	0.800
**Behavioural determinants**	Number of sex partners in the previous 6 months						
	None	ref.	-	-	ref.	-	-
	1	1.58	0.27;9.32	0.611	***1.05***	0.11;10.10	0.966
	2 to 6	3.74	0.69;20.44	0.128	1.76	0.21;14.85	0.352
	7 to 15	4.38	0.74;25.86	0.103	2.76	0.32;23.47	0.306
	More than 15	3.57	0.60;21.13	0.161	3.07	0.36;26.28	0.602
	HIV status						
	HIV-negative	ref.	-	-	ref.	-	-
	HIV-positive	4254481.23	0.00-inf	0.826	1.24	0.47;3.28	0.657
	HIV status unknown or not disclosed	0.13	0.03;1.27	0.102	0.95	0.19;4.65	0.949
	PrEP use status						
	Current PrEP users	ref.	-	-	ref.	-	-
	PrEP naïve or PrEP discontinued	0.58	0.28;1.19	0.134	0.74	0.43;1.36	0.266
	Any type of substance use in the previous 6 months						
	Never	ref.	-	-	ref.	-	-
	Ever	1.07	0.36;3.18	0.902	1.39	0.74;2.58	0.302
	Recreational drugs use in the previous 6 months [1]						
	Never	ref.	-	-	ref.	-	-
	Ever	*1.62*	0.76;3.46	0.210	*0.57*	0.25;1.32	0.194
	Chemsex in the previous 6 months [2]						

Never	ref.	-	-				ref.	-	-
Ever	0.69	0.33;1.43	0.322				0.85	0.52;1.39	0.517
Poppers use in the previous 6 months									
Never	ref.	-	-	ref.	-	-	ref.	-	-
Ever	2.02	1.01;4.05	0.047	1.44	0.67;3.12	0.355	0.93	0.58;1.48	0.762
Erectile dysfunction medication use in the previous 6 months [3]									
Never	ref.	-	-				ref.	-	-
Ever	0.96	0.48;1.88	0.886				0.96	0.60;1.54	0.854
Alcohol use in the previous 6 months									
Never	ref.	-	-				ref.	-	-
Ever	0.88	0.39;2.00	0.766				1.63	0.89;2.95	0.111
Visited a gay sauna in the previous 6 months									
Never	ref.	-	-				ref.	-	-
Ever	1.06	0.52;2.15	0.878				1,01	0.66;152	0.965
Visited a darkroom in the previous 6 months									
Never	ref.	v	-				ref.	-	-
Ever	0.85	0.43;1.68	0.634				0.81	0.50;1.32	0.254
Visited a circuit party in the previous 6 months									
Never	ref.	-	-				ref.	-	-
Ever	1.07	0.50;2.28	0.871				0.81	0.48;1.38	0.440
Visited a pride event in the previous 6 months									
Never	ref.	-	-				ref.	-	-
Ever	1.12	0.57;2.21	0.746				1.14	0.72;1.83	0.567
Visited a gay dance club in the previous 6 months									

	Never	ref.	-	-				ref.	-	-			
	Ever	0.89	0.43;1.83	0.754				1.48	0.89;2.46	0.136			
	Attended private sex parties in the previous 6 months												
	Never	ref.	-	-				ref.	-	-			
	Ever	1.16	0.54;2.47	0.709				1.12	0.68;1.85	0.662			
	Visited fetish events/fairs in the previous 6 months												
	Never	ref.	-	-				ref.	-	-			
	Ever	0.86	0.39;1.89	0.700				1.37	0.79;2.35	0.260			
**Psycho-social determinants**	Knowing anybody who has/had monkeypox												
	No	ref.	-	-	ref.	-	-	ref.	-	-			
	Yes	3.97	0.93;16.91	0.062	2.52	0.53;11.90	0.243	1.12	0.61;2.05	0.724			
	Concern about being infected by monkeypox*	2.32	1.68;3.20	<0.001	1.95	1.32;2.88	<0.001	0.87	0.72;1.05	0.151			
	Perceived risk of being infected by monkeypox*	2.21	1.55;3.16	<0.001	1.29	0.82;2.01	0.275	0.96	0.77;1.19	0.692			
	Perceived problematic consequences of monkeypox*	1.37	0.99;1.87	0.0532	1.26	0.89;1.78	0.192	0.80	0.64;1.01	0.062	0.80	0.64;1.01	0.063

Notes: [1] I use substances recreationally (for example THC, MDMA, ecstasy, etc). [2] I use substances in the context of sex (for example crystal meth/tina, GHB, ketamine etc.). [3] I use erectile dysfunction medication (for example Viagra, Kamagra). * indicates variable with a 1−5 Likert scale, with 1 = extremely unlikely and 5 = extremely likely)
